# Macroscopic Stress-Strain Response and Strain-Localization Behavior of Biopolymer-Treated Soil

**DOI:** 10.3390/polym14050997

**Published:** 2022-02-28

**Authors:** Antonio Soldo, Victor Aguilar, Marta Miletić

**Affiliations:** 1 Department of Civil and Environmetnal Engineering, Auburn University, Auburn, AL 36849-5337, USA; azs0196@auburn.edu; 2 Facultad de Ingenieria y Tecnologia, Universidad San Sebastian, Lientur 1457, Concepcion 4080871, Chile; victor.aguilar@uss.cl; 3 Department of Civil, Construction, and Environmental Engineering, San Diego State University, San Diego, CA 92182-1324, USA

**Keywords:** biopolymers, strain localization, green geotechnics, image processing, Drucker–Prager

## Abstract

The enhancement of soil engineering properties with biopolymers has been shown recently as a viable and environmentally benign alternative to cement and chemical stabilization. Interest in biopolymer-treated soil is evident from the upsurge of related research activities in the last five years, most of which have been experimental in nature. However, biopolymers have not yet found their way into engineering practice. One of the reasons for this may be the absence of computational models that would allow engineers to incorporate biopolymer-treated soil into their designs. Therefore, the main goal of this study is to numerically capture a macroscopic stress-strain response and investigate the effect of biopolymers on the onset of strain localization. Several diagnostic strain-localization analyses were conducted, thus providing strain and stress levels at the onset of strain localization, along with the orientations of the deformation band. Several unconfined compression and triaxial tests on the plain and biopolymer-treated soils were modeled. Results showed that biopolymers significantly improved the mechanical behavior of the soil and affected the onset of strain localization. The numerical results were confirmed by the digital image analysis of the unconfined compression tests. Digital image processing successfully captured high strain concentrations, which tended to occur close to the peak stress.

## 1. Introduction

Due to the rapid urbanization of cities and the growth of the human population, soil-improvement methods are of increasing importance because of the need to construct on the soft and complicated ground in very adverse and hostile surroundings [1,2,3,4]. The primary method that is conventionally applied to enhance the engineering properties of the soil is chemical treatment, and one of the most commonly used chemical-stabilization agents is cement. Even though it is effective and cost-efficient, cement has several adverse effects on the environment. For instance, it can initiate the formation of heat islands, contaminate underground water, eradicate vegetation, prevent vegetation growth, etc. However, the most severe environmental impact is the production of carbon dioxide (CO_2_) during the production of cement. The estimations are that the cement-production industry has contributed to more than six percent of the world’s CO_2_ emissions in recent years [5]. Therefore, the need to replace environmentally harmful materials, such as cement, is in high demand. 

One of the eco-friendly solutions is microbial-induced carbonate precipitation (MICP). MICP is a biological approach that requires the presence of a large microbial community in the coarse-grained soil. The reactions between microorganisms and soil particles create a cementitious bond that improves soil properties. MICP proved to be effective in increasing the strength and load-bearing capacity of soil [3,6,7,8,9]. However, this approach comes with certain drawbacks. It can result in the generation of effluent ammonia, and it can only be implemented in coarse-grained soil. The reason for the latter is that the pores of fine-grained soil are very small and hence not suitable for the habitation of microbes [4]. 

Another eco-friendly approach for soil stabilization is the utilization of biopolymers. Biopolymers are naturally made polymers extracted from plants, shells, fungi, and yeast. They have been used in the food industry, the cosmetic industry, medicine, and agriculture [10,11,12,13]. Since many of them are known as being harmless and edible, they can be considered as eco-friendly agents for soil treatment [7]. Their advantage over the MICP treatment is that they can be used in both fine- and coarse-grained soils, and they do not generate effluent ammonia [14,15]. Up to now, several research studies have shown the positive biopolymer effect on soil-strength improvement, permeability reduction, and soil-collapsibility decrease [14,16,17,18,19,20,21].

The vast majority of the research on biopolymer-treated soil has been experimental in nature. One of the reasons why the biopolymer treatment of soil has not found its way into civil-engineering practice could be the absence of computational models that would allow engineers to incorporate biopolymer-treated soil into their designs. Some of the research that conducted numerical modeling is by Ayeldeen et al. [16]. They created a finite-element model to investigate the behavior of the treated collapsible soil after and before water immersion. The treated and untreated soils were simulated by Mohr–Coulomb’s criterion with an associated flow rule. The commercially available finite-element software Plaxis 2D was used. The results of the numerical analysis showed that treating the soil with a biopolymer would increase the soil-bearing capacity and reduce soil settlement during and after saturation. The results of the numerical analysis on untreated soils were in close agreement with the results of the in situ plate-load tests. 

Another example of numerical modeling of the biopolymer-treated soil was presented by Chen et al. [22]. In their experimental and numerical research, the surface strength of biopolymer-treated mine tailings, made of finely ground rock, was investigated. They conducted experimental research on the biopolymer effect on the penetration force of a cylindrical penetrometer. In the numerical part of their study, they used the discrete-element method to simulate the penetration test on the mine tailings stabilized with biopolymer solutions. Their numerical analysis, which was conducted in PFC3D v4.0 using the parallel-bond model, showed coinciding results with their experimental analysis. The numerical simulations showed an increase in the tensile and shear strengths with higher biopolymer concentration, indicating that the higher concentration of biopolymer causes greater inter-particle bonding. 

To the best knowledge of the authors, no previous attempts have been made towards conducting a diagnostic strain-localization analysis in biopolymer-treated soil. Strain localization is a characteristic of elastic-plastic materials that indicates the onset of narrow deformation bands. It is characterized by a jump in strain rate and is followed by a reduction in load-carrying capacity, which often indicates an imminent failure of the materials and structures. Thus, the main goal of this study was to numerically capture and experimentally validate a macroscopic stress-strain response and predict the onset of strain localization in elastic-plastic biopolymer-treated soil. In particular, this study focused on soil treated with three types of biopolymers that were modeled by the linear Drucker-Prager model. A combined numerical-analytical algorithm that can capture the stress-strain response and the inception of strain localization in biopolymer-treated soil was implemented. Actual-unconfined compression and unconsolidated-undrained tests, which were performed on plain and biopolymer-treated soil, were modeled. The analyses presented herein allowed an improved characterization of the biopolymer effect on the failure initiation by providing the stress and strain levels at the onset of strain localization, as well as the orientations of the accompanying discontinuities and corresponding strain-localization modes. Ultimately, the diagnostic strain-localization analyses provided a quantitative measure of the biopolymer contribution toward the increased resilience and toughness of these important and environmentally friendly materials. 

Following the introduction in this section, a detailed description of the experimental program is provided. This is followed by a description of the conditions for the onset of discontinuous bifurcations and the application of the Drucker–Prager model. Finally, the calibration of constitutive models, stress-strain responses, and predictions for the onset of strain localization are presented. A summary of the major findings is provided at the end.

## 2. Experimental Research

### 2.1. Soil

Two types of sand were used as a base material in this study: silty sand (All-Purpose Sand—Quikrete) and pure sand (Premium Play Sand—Quikrete). Both sand types were classified following the ASTM D6913-17 [23] and ASTM D4318-17 [24] standards. The silty sand had 39% fine particles with the liquid limit, plastic limit, and the index of plasticity being 49, 29, and 20, respectively. Therefore, according to the Unified Soil-Classification System, fine particles were classified as silt with low plasticity, and the overall classification of this soil was SM (silty sand).

The pure sand was obtained from a site close to Destin, Okaloosa County, Florida, USA. It was mostly made of quartz and was highly uniform. Fine-particle content was almost non-existent. The coefficient of uniformity and the coefficient of curvature that were calculated from the grain-distribution curve were 1.46 and 0.93, respectively. Since the presence of gravel and fine particles was practically non-existent, USCS classifies this sand as SP (poorly graded sand). This type of sand was investigated only for the image-processing segment of this research.

### 2.2. Biopolymers

#### 2.2.1. Xanthan Gum

Xanthan gum (XG) is a biopolymer created by the fermentation of a carbohydrate-source medium such as glucose. Xanthan gum was named after the bacterium that induces the process of fermentation, *Xanthomonas campestris*. Xanthan gum is easily dissolved in hot and cold water. Solutions containing Xanthan gum are non-Newtonian with high pseudoplasticity. At low shearing rates, the xanthan gum chains are in a state of rest and bound by hydrogen bonds. When increasing the shear rate, the bonds are reduced, which leads to lower viscosity. Due to its fast interaction with water, rapid agitation and mixing are needed to efficiently dissolve xanthan gum in water. The chains of xanthan gum remain stable over wide ranges of pH values. Therefore, it can be successfully implemented in cleaning products as well as acidic food additives [10]. Otherwise, it can be found in the cosmetic, agriculture, and oil-drilling industries [10]. Commercially available Bob’s Red Mill Xanthan Gum (Milwaukie, OR, USA) was used in this study. 

#### 2.2.2. Guar Gum

Guar gum (GG) is a biopolymer extracted from *Cyamopsis tetragonoloba* that is commonly known as guar. Unlike the majority of plant-based gums, guar gum does not have any uronic acid in its molecular structure. Additionally, it has a high molecular weight when compared with other naturally occurring water-soluble polysaccharides. It is worth mentioning that guar-gum formations are stable over a broad range of pH. Therefore, it can be dissolved in water. Even small concentrations of guar can significantly increase the viscosity of the solution it is mixed in. Some of its applications can be found in the cosmetic, food, agriculture, and oil-drilling industries [13]. In this study, commercially available Bob’s Red Mill Guar Gum (Milwaukie, OR, USA) was used, and the study complies with local and national regulations. 

#### 2.2.3. Beta-Glucan

Beta-glucan (BG) is a biopolymer that consists of glucose molecules. It is extracted from the cells of yeast, fungi, some types of bacteria, and certain types of cereals. The molecular structure of beta-glucans depends on the source they were extracted from. Beta-glucan molecules can vary in the kinds of linkages, branching, molecular weight, solubility, and polymer charge [25]. The beta-glucan used in this study was beta-glucan 1.3/1.6. The extraction process of beta-glucan depends on the parent source and the molecular bonds of the polymer. Therefore, an appropriate solving agent must be selected. Molecular structures of beta-glucan that are held loosely outside the cells can be extracted with hot water. Beta-glucan molecules that are held tightly to the cell walls can be released by hot alkali. Beta-glucan, in its powder form, can be dissolved in hot and cold water, which results in forming a gelatinous solution [26]. Beta-glucans are heavily investigated in medicine for health improvement [25,26]. Beta-glucan 1.3/1.6 produced by the Bulk Supplements company (Henderson, NV, USA) was used in this research study. 

#### 2.2.4. Specimen Preparation

All specimens were prepared by the dry mixing of biopolymer and silty sand. The biopolymer concentrations that were used were 0, 1, 2, and 4% with respect to the mass of the soil, where 0% represents the plain soil with no additives. After the biopolymer was uniformly mixed with soil, water was sprayed up to 16.5% of the soil mass. After the water was homogeneously added to the biopolymer-soil mixture, the biopolymer-soil was compacted in the molds that were intended for the unconfined compression, splitting-tensile, and triaxial tests. 

The mold for the specimens that were tested for unconfined compression had a diameter of 3.3 cm and a height of 7.1 cm. Additionally, several cube specimens with dimensions of 5 × 5 × 5 cm were made in order to monitor the development of the strain and to detect the inception of strain localization during the unconfined compression test by means of digital image correlation. The cube specimens were chosen because of the flat surface of the sides of the cube. Specimens for the triaxial test had a mold with a diameter of 7 cm and a height of 14 cm. Furthermore, specimens for the splitting-tensile-strength test were made with the intention of using them for the calibration of the numerical model. The mold that was used to make the specimens for the splitting-tensile-strength test had a diameter and height of 3.5 and 1.8 cm, respectively. After preparation, specimens were extruded from the molds and left to cure for five days (specimens for the unconfined compression and splitting-tensile test) of time and seven days (specimens for the triaxial test). The reason for different curing times is the fact that the triaxial specimens were larger and required more curing time to achieve their full strength.

#### 2.2.5. Mechanical Testing

For determination of strength in the unconfined compression test, the axial load was applied with a strain rate of 1.5 %/min, as per ASTM D2166 [27]. The compressive strength of the specimen was computed by dividing the maximum load attained during the test by the corresponding cross-sectional area of the specimen. 

The unconsolidated-undrained test was performed in accordance with ASTM D2850-15 [28]. The specimens were placed in a plexiglass chamber under the confining pressure of 100 kPa. During the shearing stage of the test, the axial strain was applied under the rate of 0.7 %/min. 

Splitting-tensile tests were performed following general procedures described in the ASTM D3967-16 [29] but with a modified apparatus to accommodate small specimen sizes and relatively low loads. The load was applied at the constant strain rate of 1.5 %/min. It must be noted that the splitting-tensile test does not give direct soil-tensile properties. Thus, the tensile strength was back-calculated from the maximum compressive load and the dimension of the specimens using the following formula [30]:

(1) $$\sigma_{T}=\frac{2P}{\pi LD}$$

where *σ_T_* is tensile strength; *P* is a compressive force at failure; *D* is the diameter of the specimen; *L* is the specimen thickness.

#### 2.2.6. Digital Image Acquisition and Processing

The digital image-processing technique was used to monitor the development of the strain component and detect the inception of strain localization. The cube specimens were chosen because of the flat surface of the sides of the cube. The flat surfaces of the cube specimens were appropriate for the image-processing software (GOM Correlate, Braunschweig, Germany) [31], which can record the strain development in pressure-sensitive materials. 

Since the quality and accuracy of the image processing rely on a high-contrast random pattern, a distinctive speckle pattern was applied to the surface of the specimen with a colored marker. The digital images of the soil samples were continuously recorded at a constant position (20 cm from the center of the specimen) for image consistency and uniformity. The resolution of the camera was 12 MP, and it was able to capture 30 frames per second. 

The image-processing software splits the recording into a series of images. The applied pattern is recognized by the software and isolated as a surface of observation. That surface is transformed into a group of pixels. In this study, the surface of interest (45 × 45 mm) was transformed into approximately 570 × 570 pixels. The position of each pixel and the distance from the surrounding pixels are tracked through the software. Each picture is compared to the previous one, therefore the software can detect the change in the pixel position. Following that technique, the software can detect and quantify displacement and strains that occur in the initial recording. Furthermore, the unconfined compression apparatus stores timestamps throughout the test, while the image-processing software takes the time steps from the video recording. Therefore, a correlation between the experimental results and the image-processing software could be drawn.

## 3. Numerical Modeling

### 3.1. Stress-Strain Relationship

The biopolymer-treated soil was assumed to be an elastic-plastic material experiencing an infinitesimal strain and obeying a general non-associative flow rule. The normal components of stress and strain tensors are positive in tension herein. The macroscopic stress-strain relationship for plastic loading is given by [32,33]:

(2) $${\dot{\sigma }}_{ij}=D_{ijkl}^{e}({\dot{\varepsilon }}_{kl}-{\dot{\varepsilon }}_{kl}^{p})$$

where subscript notation represents the order of the tensor, superscripts indicate elastic (*e*), plastic (*p*) or elastic-plastic (*ep*) component, “∙” over a symbol represents “rate” (time derivative). Therefore, ${\dot{\sigma }}_{ij}$, ${\dot{\varepsilon }}_{ij}$, and ${\dot{\varepsilon }}_{ij}^{p}$ are the rates of a second-order Cauchy-stress tensor, infinitesimal-strain tensor, and infinitesimal-plastic-strain tensor, respectively. $D_{ijkl}^{e}$ is the corresponding elastic-stiffness-moduli tensor of the biopolymer-treated soil, and is given by [33]:

(3) $$D_{ijkl}^{e}=\mu (\delta_{ik}\delta_{jl}+\delta_{jk}\delta_{il})+\lambda \delta_{ij}\delta_{kl}$$

and *δ_ij_* is the Kronecker delta, and μ,λ are Lamé’s constants of the composite.

The two-invariant yield function (*F*) was used to describe the plastic behavior of a pressure-sensitive cementitious biopolymer-treated soil. It is defined as:

(4) $$F=F(\sigma_{ij},\kappa )$$

where κ is a plastic-hardening variable. 

The plastic flow rule and hardening law are respectively given by:

(5) $${\dot{\varepsilon }}_{ij}^{p}=\dot{\lambda }\frac{\partial G}{\partial \sigma_{ij}}$$

and

(6) $$\dot{\kappa }=h\left({\dot{\varepsilon }}_{ij}^{p}\right)=\dot{\lambda }h\left(\frac{\partial G}{\partial \sigma_{ij}}\right)$$

where $\dot{\lambda }\geq 0$ is a plastic multiplier, $h({\dot{\varepsilon }}_{ij}^{p})$ is the first-order homogeneous, generally nonlinear function, and *G* is a plastic-potential function. The non-associated flow rule was used to more realistically model the behavior of the treated pressure-sensitive materials such as biopolymer soils [34,35].

The plastic multiplier $\dot{\lambda }$ is obtained from the consistency condition in plastic loading, as:

(7) $$\dot{\lambda }=\frac{f_{ij}D_{{}_{ijkl}}^{e}{\dot{\varepsilon }}_{kl}}{H+f_{ij}D_{{}_{ijkl}}^{e}g_{kl}}$$

where gradients of the yield function and plastic potential are denoted by *f_ij_* and *g_ij_*, respectively. The actual-hardening modulus *H* is given by:

(8) $$H=-\frac{\partial F}{\partial \kappa }h(g_{ij})$$

It is positive, negative, or zero for hardening, softening, or perfect plasticity, respectively.

By combining Equations (2), (5) and (7), a tangent elastic–plastic-stiffness-moduli tensor $D_{ijkl}^{ep}$ is obtained as:

(9) $$D_{ijkl}^{ep}=D_{ijkl}^{e}-\frac{D_{ijmn}^{e}g_{mn}f_{pr}D_{prkl}^{e}}{H+f_{mn}D_{mnpr}^{e}g_{pr}}$$

The yield function, *F*, and rate of the plastic multiplier satisfy Kuhn–Tucker conditions as follows:

(10) $$\dot{\lambda }\geq 0,\;F(\sigma ,\kappa )\leq 0,\;\dot{\lambda }F(\sigma ,\kappa )=0$$

### 3.2. Onset of Strain Localization

The diagnostic strain-localization analysis was performed at the constitutive level. The inception of strain localization may be considered as a bifurcation problem that signifies a loss of stability of the constitutive relation governing a uniform deformation. 

It is assumed that the jump in the displacement rate along the singular surface Γ is constant, which is expressed as [33]:

(11) $${\left[{\dot{u}}_{i}\right]|}_{\Gamma }={\left({\dot{u}}_{i}{}^{+}-{\dot{u}}_{i}{}^{-}\right)|}_{\Gamma }=\mathrm{constant}$$

where ${\dot{u}}_{i}{}^{+}$ denotes the displacement rate on one side of the Γ and ${\dot{u}}_{i}{}^{-}$ the other side. 

Equation (11) generally describes the kinematics of a strong discontinuity. The special case obtained by equalizing the right-hand side of Equation (11) to zero represents a weak discontinuity. At this point, no assumptions are made about the homogeneity and variation of $\left[{\dot{u}}_{i}\right]$ in terms of the singular surface Γ. Combining Equation (11) with the kinematical definition of infinitesimal-strain rate gives the following expression for a jump in strain rate across the singular surface Γ:

(12) $${\left[{\dot{\varepsilon }}_{ij}\right]|}_{\Gamma }=\frac{1}{2}\left(z_{i}n_{j}+z_{j}n_{i}\right)$$

where *z_i_* is the eigenvector corresponding to the relevant eigenvalue problem, as discussed below, and *n_i_* is the unit vector that is normal to the surface Γ.

Furthermore, the equilibrium along the singular surface imposes the following condition on the traction rate ($\dot{t}$):

(13) $${\left[{\dot{t}}_{i}\right]|}_{\Gamma }={\left({\dot{t}}_{i}{}^{+}-{\dot{t}}_{i}{}^{-}\right)|}_{\Gamma }=0$$

Combining Equations (2), (12) and (13) leads to the classical bifurcation criterion (Rudnicki and Rice 1975) which is, given by:

(14) $$Q_{ik}z_{k}=n_{i}D_{ijkl}^{ep}n_{l}z_{k}=0$$

where $Q_{ik}$ is an acoustic tensor, also known as the characteristic-tangent-stiffness tensor. Equation (14) is based on the assumption that a so-called plastic–plastic bifurcation precedes an elastic–plastic bifurcation. In the former case, the primary and bifurcated incremental fields both correspond to plastic loading, while in the latter, only one of the incremental fields corresponds to plastic loading. It was shown [36] that the plastic–plastic bifurcation always precedes elastic-plastic bifurcation.

In order to solve for the critical amount of hardening necessary for the onset of strain localization, the following eigenvalue problem was considered:

(15) $$Q_{ik}z_{k}^{\left(j\right)}=\lambda^{\left(j\right)}Q_{ik}^{e}z_{k}^{\left(j\right)},\;j=1,2,3$$

where (*j*) indicates the direction of principal stresses. 

Nontrivial solutions of the eigenvalue problem are possible only when the acoustic tensor $Q_{ik}$ is singular. The first two eigenvalues are elastic and equal to one while the third eigenvalue is plastic, and it was given by Runesson et al. [36] as:

(16) $$\lambda^{\left(3\right)}=1-\frac{b_{i}P_{ik}^{e}a_{k}}{H+f_{mn}D_{mnpr}^{e}g_{pr}}$$

where $P_{ik}^{e}$ is the inverse of the elastic-acoustic tensor and the vectors $a_{i}$ and $b_{j}$ are defined as

(17) $$a_{i}=f_{mn}D_{mnij}^{e}n_{j},\;b_{j}=n_{i}D_{ijmn}^{e}g_{mn}$$

The corresponding eigenvector is given by the following expression:

(18) $$z_{i}^{\left(3\right)}=kP_{ij}^{e}b_{j}$$

where k is an arbitrary scalar.

By setting $\lambda^{\left(3\right)}$ from Equation (16) equal to zero, the hardening modulus *H* (*n_i_*) was obtained. Finally, the critical amount of hardening necessary for the onset of strain localization was obtained by solving the following constrained optimization problem:

(19) $$H_{cr}=\underset{n_{i}}{\max}H(n_{i}),\;\mathrm{where}\;n_{i}n_{i}=1$$

where the right side of the equation above represents the maximization of the function. The corresponding bifurcation angle *θ* represents the angle between the minor stress axis and the normal vector (*n*_1_, 0, *n*_3_). It can be determined from the expression:

(20) $$\tan^{2}\theta =\frac{n_{1}^{2}}{n_{3}^{2}}$$

Analytical solutions for *H_cr_* and corresponding critical-bifurcation angles were given by [36].

### 3.3. Application to Drucker–Prager Model

The analysis in this study was based on the linear Drucker–Prager model and the parameters of the total stress. The yield and plastic-potential functions were presented as follows:

(21) $$F_{}=\left(\frac{1}{3}\tan\beta \right)I_{1}+\sqrt{J_{2}}-\kappa$$

(22) $$G=\left(\frac{1}{3}\tan\psi \right)I_{1}+\sqrt{J_{2}}$$

The gradients of the linear Drucker–Prager yield and plastic-potential functions are given by

(23) $$f_{ij}=\frac{1}{3}\tan\beta \cdot \delta_{ij}+\frac{\sqrt{3}}{2\sqrt{J_{2}}}s_{ij}$$

(24) $$g_{ij}=\frac{1}{3}\tan\psi \cdot \delta_{ij}+\frac{\sqrt{3}}{2\sqrt{J_{2}}}s_{ij}$$

where *s_ij_* is the stress-deviator tensor; *β* is the internal-friction angle; *ψ* is the dilatancy angle; *I*_1_ and *J*_2_ are the first and second deviatoric-stress invariants, respectively.

After the critical hardening has been obtained, the onset of strain localization can be determined. The onset can be determined by comparing the values of the critical-hardening modulus with the actual-hardening modulus. The actual-hardening modulus (*H_act_*) is calculated from the response representing the actual stress and plastic strain. 

### 3.4. Numerical Simulation

The actual stress–strain response was obtained by simulating the samples by a single eight-node element modeled with 3D integration. The unconfined compression tests were conducted by applying a constant vertical-strain rate and keeping the principal stresses in the horizontal directions at zero. The unconsolidated-undrained tests were performed under a constant vertical-strain rate and applying the horizontal pressures following the experimental tests. The inception of the strain localization occurs at the moment when the actual-hardening modulus equalizes with the critical-hardening modulus.

### 3.5. Calibration of Constitutive Model

**Figure 1:**
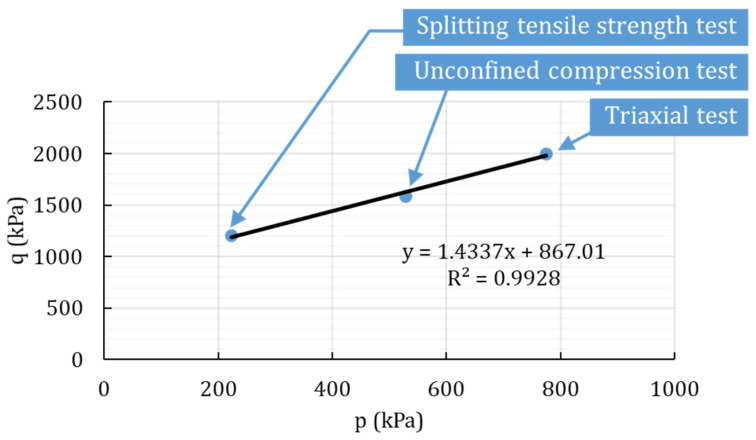
The example of the calibration procedure for the silty sand with 4% BG.

The Drucker–Prager model was calibrated against the unconfined compression, splitting-tensile-strength test, and the unconsolidated-undrained tests that were performed on plain and biopolymer-treated silty sand. The calibration example is presented in Figure 1. 

The experimental data were available for the plain soil and the soil treated with three biopolymer concentrations (1, 2, and 4%). However, only the data for the soil treated with 0, 1, and 4% were used for the calibration. The data for the soil treated with 2% biopolymers were used for the validation of the proposed model. The elastic and plastic parameters were predicted from linear interpolation of the results for the soil with 0, 1, and 4% biopolymers. To achieve the best values of the input parameters, the least-squares fit was used. The least-squares fit minimizes the relative error, and it was based on the experimental data. 

Young’s modulus was obtained for the plain and treated soil from the unconfined compression tests. Young’s modulus was calculated as the slope of the elastic portion of the stress–strain response. The calculated values of Young’s modulus for the plain and treated soil were in the range of previously reported values [37,38]. Poisson’s ratio (ν) was kept constant for all of the biopolymer-treated soil and was selected from the recommended values for different types of materials [39].

For the linear Drucker–Prager criterion used in this study, the internal friction angle (*β*) and cohesion (d) were taken from the linear yield surface in the p–q stress plane (Figure 1). The cohesion for the samples with 2% biopolymer was calculated from the predicted friction angle and the initial stress level of the predicted hardening. The plasticity models for the non-associated flow required a value for the dilatation angle (*ψ*) that was kept constant for all of the biopolymer-treated soil and was assumed based on suggested values [40]. Table 1 summarizes the material properties used for the proposed model.

**Figure 2:**
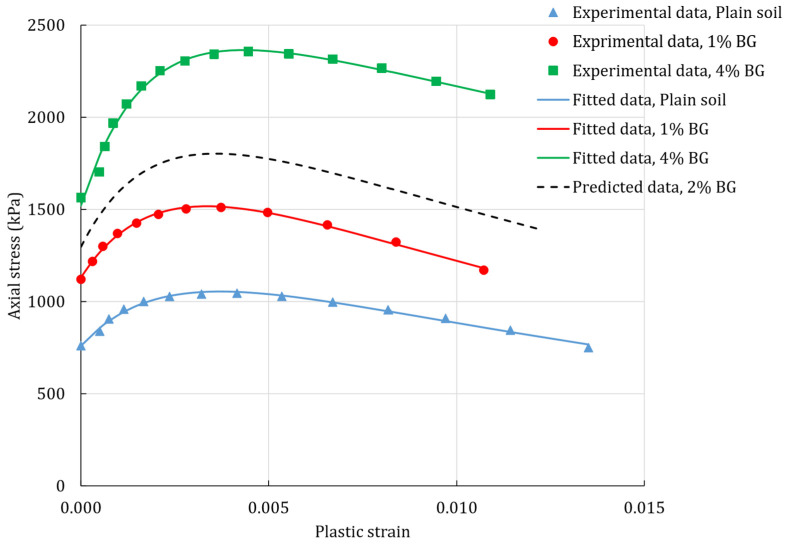
Hardening response for the unconfined compression test for silty sand treated with BG.

The isotropic non-linear hardening was used in all of the test simulations and was implemented into the model in a tabular form. The hardening was obtained from the averaged values of the experimental tests (unconfined compression). The plastic strains were calculated by subtracting the elastic-strain components from the amount of total strain. Prior to that, the elastic-strain components were calculated by dividing the axial-stress response with the modulus of elasticity. The example of actual and predicted hardening responses for the unconfined compression are presented in Figure 2. The hardening curve for the silty sand with 2% BG was interpolated from the parameters of the equations fitting the experimental data.

## 4. Results

### 4.1. Unconfined Compression Test

**Figure 3:**
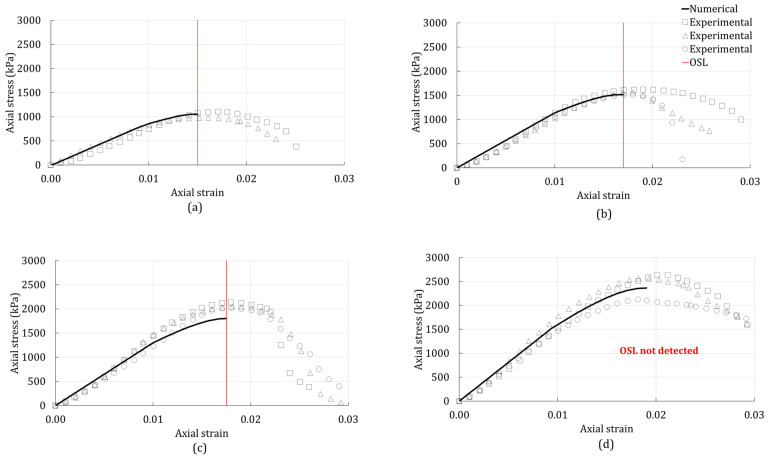
Unconfined compression-test results of the silty sand with (**a**) 0% additives; (**b**) 1% BG; (**c**) 2% BG; (**d**) 4% BG.

**Figure 4:**
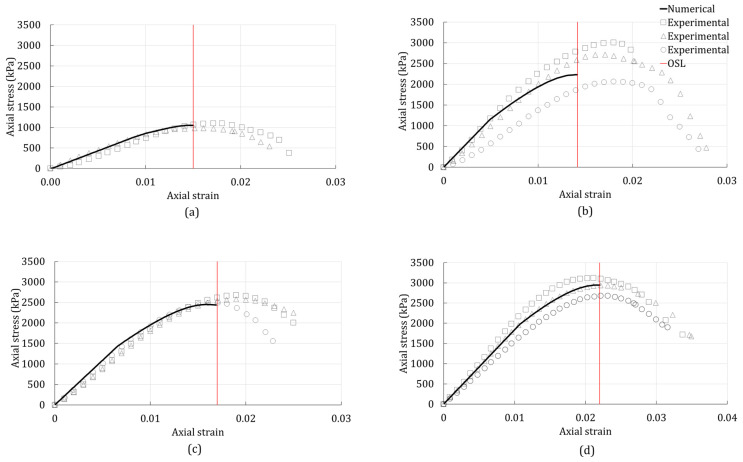
Unconfined compression-test results of the silty sand with (**a**) 0% additives; (**b**) 1% GG; (**c**) 2% GG; (**d**) 4% GG.

**Figure 5:**
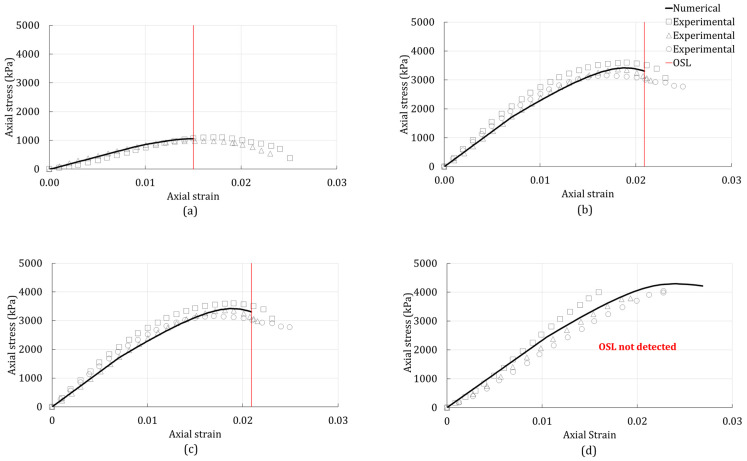
Unconfined compression-test results of the silty sand with (**a**) 0% additives; (**b**) 1% XG; (**c**) 2% XG; (**d**) 4% XG.

The linear Drucker–Prager model was used to simulate the 3D stress state of the unconfined compression tests. A comparison between the numerically predicted responses and experimentally observed responses for the unconfined compression test are presented in Figure 3, Figure 4 and Figure 5. The stress-strain responses of specimens with 0, 1, and 4% were used for the calibration of the model, while the specimens with 2% were used for validation. 

Figure 3 shows the numerical response (solid line) and experimental data (scatter data) of the unconfined compression test of the BG-treated silty sand. For comparison, the same results of the plain silty sand are shown in Figure 3a). From Figure 3, it can be seen that the onset of strain localization (OSL) of the silty sand increased with the addition of BG. Furthermore, it can be seen that the peak stress also increased with the biopolymer addition. Figure 3a,b,d show a relatively better match between the experimental data and the numerical response when compared with Figure 3c. That is because the numerical response in Figure 3a,b,d were based on the calibrated material properties, whereas the numerical response in Figure 3c was obtained from the predicted material properties. The predicted model could not detect the OSL for the silty sand treated with 4% BG because the actual critical hardening never equalized with the calculated critical hardening. Hence, there is no OSL (red line) shown in Figure 3d.

In Figure 4, the numerical response and experimental data of the unconfined compression test of the GG-treated silty sand can be seen. Additionally, the experimental and numerical results of the treated silty sand were compared with the results of the plain silty sand (Figure 4a). It can be seen that the peak stress increased with the increase of the GG concentration. Figure 4 shows that the OSL of the silty sand increased with the addition of GG. The exception is the concentration of 1% GG (Figure 4b). Even though the peak stress of the silty sand increased with the addition of 1% GG, the OSL occurred at the lower strain level for 1% GG. The reason behind this is that the onset of strain localization depended on the yield stress and the peak stress, but it was primarily governed by the hardening response. Furthermore, the numerical response of the silty sand with 2% GG showed a relatively good match with the experimental data (Figure 4c). 

Figure 5 shows the experimental data and the numerical response of the unconfined compression test of the XG-treated silty sand. Figure 5 shows that the OSL of the silty sand was postponed with the addition of XG. Additionally, it can be seen that the peak stress also increased with the increase in XG. The numerical response of the silty sand treated with 2% XG showed a relatively good correspondence with the experimental data even though it was based on the predicted material properties. The predicted model could not detect the OSL for the silty sand treated with 4% XG because the actual critical hardening did not rise to the level of the calculated critical hardening. Therefore, as earlier, it is not shown in Figure 5d. 

**Figure 6:**
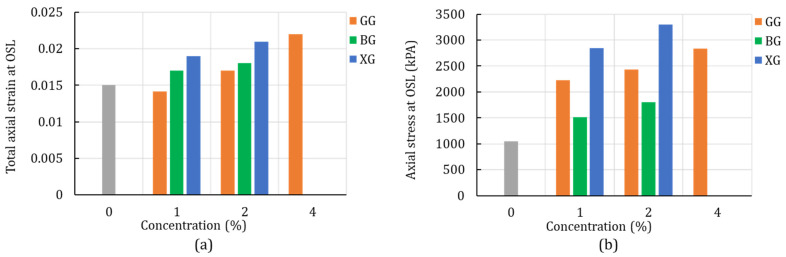
Unconfined compression-test results: (**a**) axial strain at OSL and (**b**) axial stress at OSL for the unconfined compression test of silty sand.

Figure 6 summarizes the level of the total axial strain (Figure 6a) and the axial stress at the OSL (Figure 6b). It is evident that the stress at the OSL increased with the increase in biopolymer concentration, and that the level of total strain increased for most of the biopolymer concentrations. 

**Figure 7:**
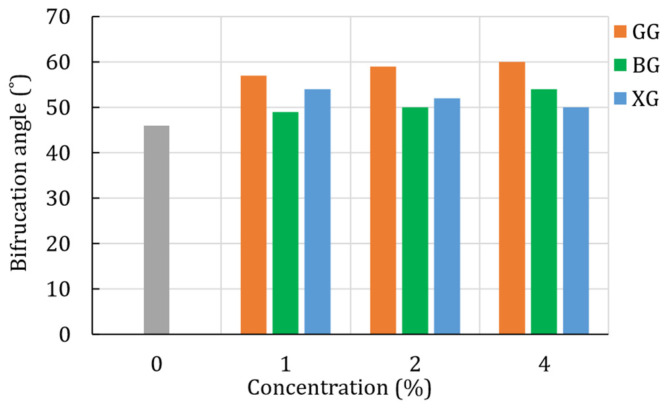
Bifurcation angle for the plain and biopolymer-treated silty sand (unconfined compression test).

The bifurcation angle is represented by the angle between the minor stress axis (x_3_) and the unit vector (n) that lays in the x_1_, x_3_ plane [36]. From Figure 7, it can be seen that the addition of the biopolymers altered the bifurcation angle. In particular, the results show that the bifurcation angle kept increasing with the increase in the GG and BG concentrations. For the XG-treated soil, the highest bifurcation angle was achieved for the concentration of 1%, and it kept decreasing with the increase in XG concentration. That phenomenon can be related to the increase in the ductility of the material with the increase in the XG concentration. However, in all cases of the treated soil, the bifurcation angle was higher than that of the plain soil. In general, the increase in the bifurcation angle results in a steeper deformation bend that changes the failure behavior.

### 4.2. Unconsolidated-Undrained Triaxial Test

**Figure 8:**
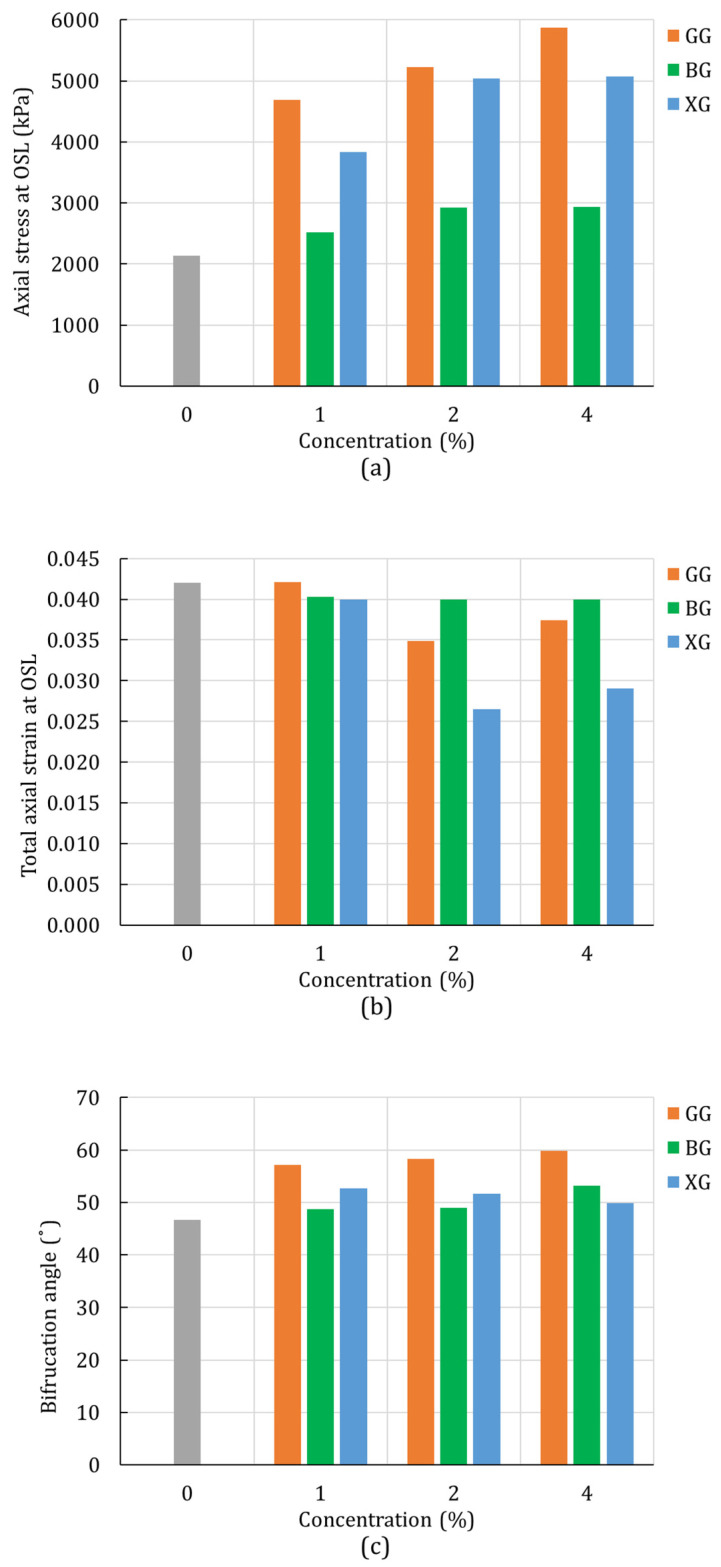
Unconsolidated-undrained triaxial test results of silty sand (**a**) axial stress at OSL; (**b**) axial strain at OSL; (**c**) bifurcation angle.

The linear Drucker–Prager model was also used to simulate the 3D stress state of the unconsolidated-undrained triaxial test. The experimental stress-strain response was compared with the numerical response. The onset of strain localization was considered when the actual hardening equalized with the critical hardening. The results are summarized in Figure 8. The level of the stress at OSL increased with the increase of the biopolymer concentration (Figure 8a). However, Figure 8 shows a trend of the decreasing level of strain at the OSL with the increase in biopolymer concentration. The reason for this behavior is the fact that the onset of strain localization depends on the yield stress and the hardening response. These results indicate that the biopolymer-treated soil had a more brittle behavior than the plain soil at higher confining pressures.

The change in the bifurcation angle of triaxial specimens (Figure 8c) showed a similar response, as seen in the unconfined compression test. For all biopolymer types and biopolymer concentrations, the treated soil had a higher bifurcation angle than the plain soil. Additionally, the bifurcation angle kept increasing with the concentration increase of GG and BG. For the XG-treated soil, the highest bifurcation angle was achieved for the concentration of 1%, and it kept decreasing for the higher concentrations of XG.

**Figure 9:**
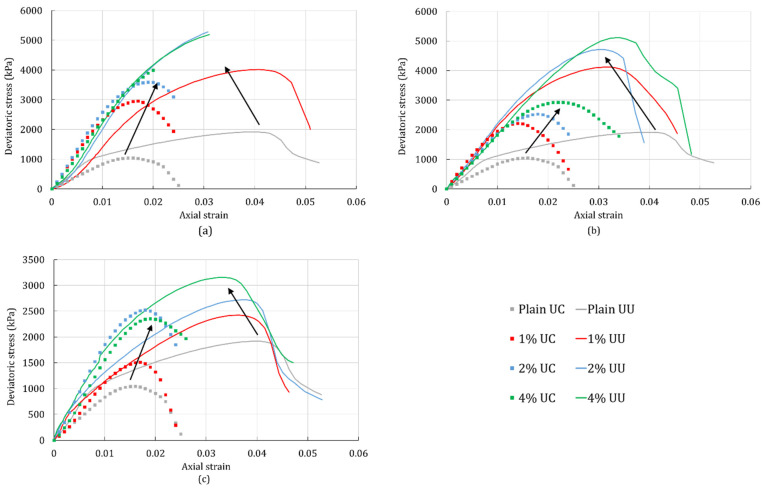
Comparing unconfined compression test (UC) with the triaxial test (UU) for the plain silty sand and silty sand treated with (**a**) XG; (**b**) GG; (**c**) BG. The arrows indicate the shift of the stress-strain curves.

Figure 9 compares the stress-strain responses of the unconfined compression tests and the triaxial tests of the silty sand. As described previously, the onset of strain localization occurs close to the peak of the stress-strain curves. Comparing the peaks of the unconfined compression- and triaxial-test curves, it can be observed that the peaks occur at a higher stress-strain level for the triaxial tests. The reason for this is the applied confinement pressure in the triaxial test. Schnaid et al. [41] demonstrated that the increase in confinement pressure increases the level of the peak stress in the cemented sand and it postpones the level of strain at which it occurs. Furthermore, the peak stress tends to increase with the increase in biopolymer concentration. Observing only the results of the unconfined compression tests, represented by scattered data, it can be seen that biopolymers tend to increase the level of the peak stress and the level of strain at the peak (represented by the arrow in Figure 9). The triaxial test results, represented by solid lines, also show that the level of the peak stress increases with the addition of biopolymers. However, the peak occurs at a lower strain level for the treated silty sand than for the plain silty sand. Furthermore, the level of strain tends to decrease with the increase in biopolymer concentration (represented by the arrow in Figure 9). Similar results were demonstrated in previous research on stabilized soil [41,42]. This phenomenon is likely related to the ductility of the specimens. The plain soil in the triaxial test demonstrated a more ductile behavior than the treated specimens. Cemented soil can have an abrupt post-peak failure in the triaxial test [43] that indicates the brittleness of the treated soil. It is also more prominent in sand with few or no fine particles. 

### 4.3. Digital Image Correlation

**Figure 10:**
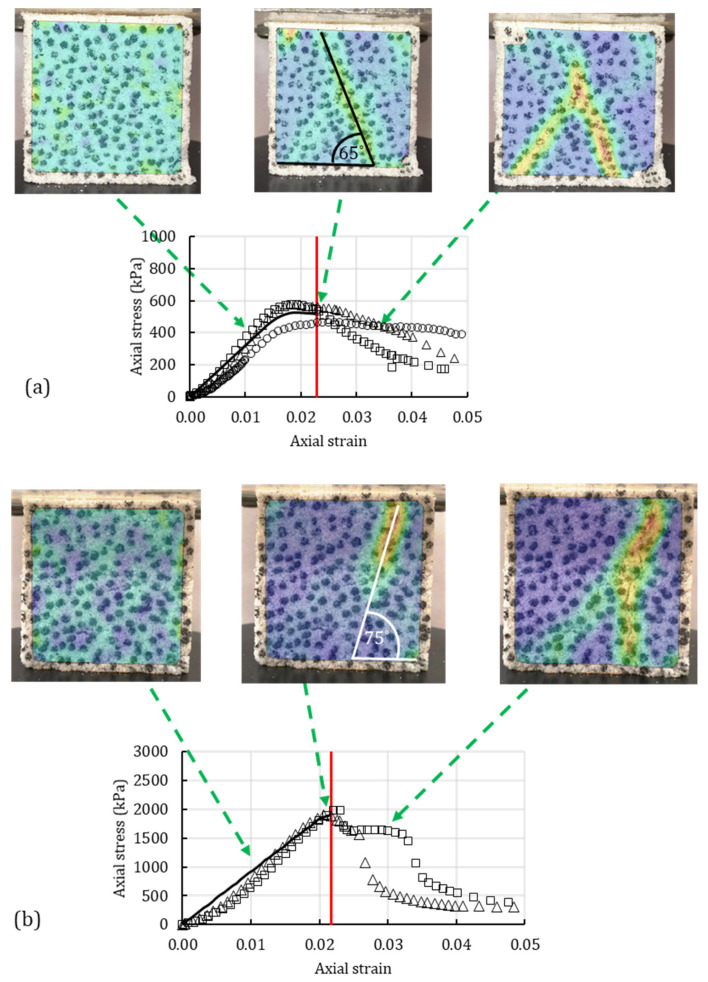
Image processing of strain localization for sand with (**a**) 0.5% XG and (**b**) 1% XG under unconfined compression test compared with the numerically obtained OSL (red line) and experimental data.

**Figure 11:**
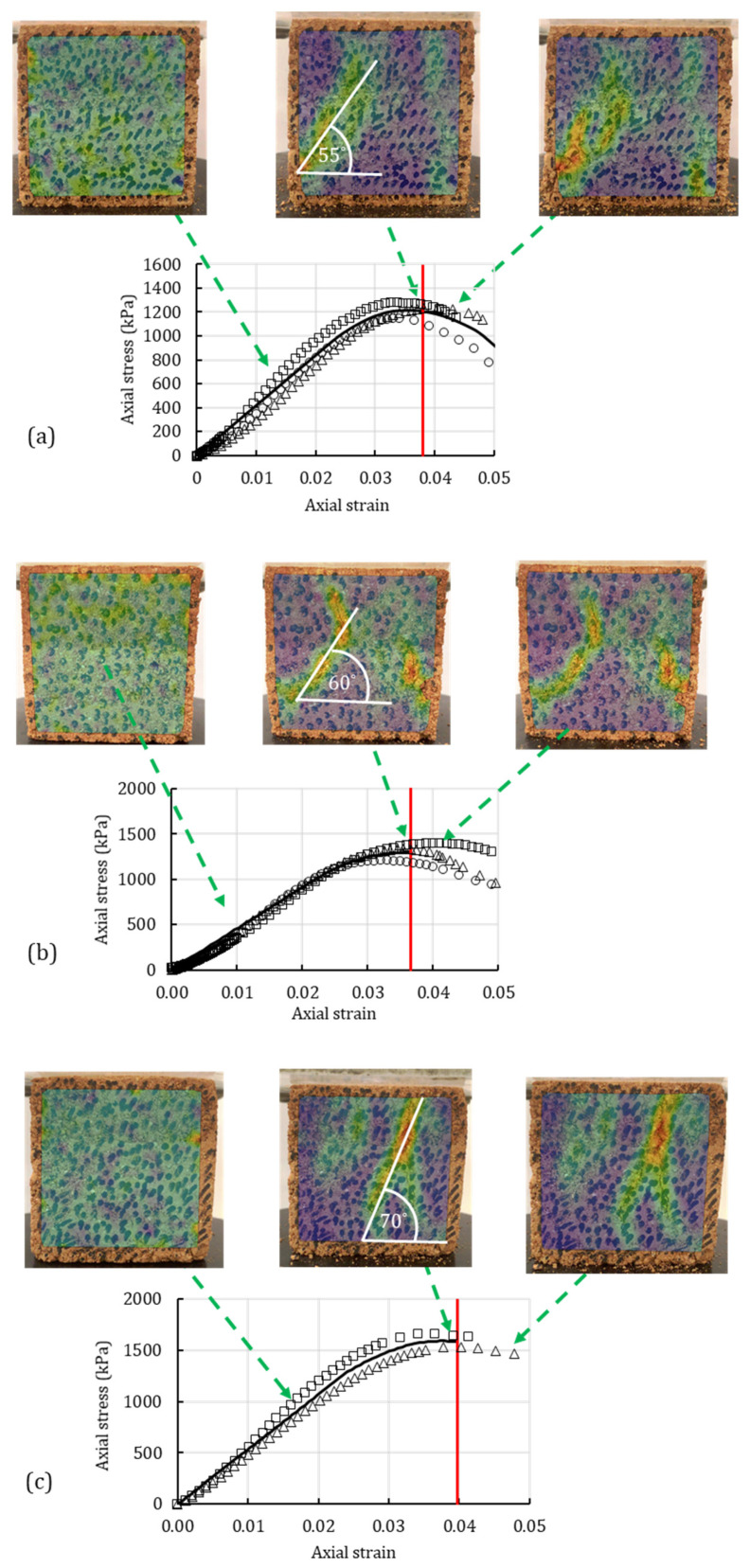
Image processing of strain localization for the silty sand with (**a**) no additives; (**b**) 0.5% XG; (**c**) 1% GG under unconfined compression test compared with the numerically obtained OSL (red line) and experimental data.

Figure 10 and Figure 11 combine the experimental response, numerical response, and image-processing results for clean sand and silty sand, respectively. The experimental responses of the unconfined compression tests are presented by markers in Figure 10 and Figure 11. The solid dark and red lines represent the numerically obtained stress-strain curve and the OSL, respectively. 

The heat maps in Figure 10 and Figure 11 are presented as overlays on the soil surface and show the strain concentration and strain propagation on the cube samples. Three heat maps represent three different timestamps when the heat map was generated. The first image represents the early stage of the unconfined compression with no visible strain localization. The second image corresponds to the OSL from the analytical-numerical algorithm. The third image represents the post-peak strain propagation. The arrows in the figures represent the stress-strain level at which the heat map was captured. 

Figure 10a represents the sand treated with 0.5% XG, whereas Figure 10b represents the sand treated with 1% XG. Comparing the onset of strain localization in the sands with different XG concentrations, it can be seen that OSL occurs at almost the same strain level; in fact, it is moderately lower for the sand with 1% XG. However, the peak stress was higher for the specimen treated with 1% XG. Shortly after reaching the peak stress, the stress dropped more abruptly for the specimen with 1% XG because of the increased brittleness. 

The heat maps in Figure 10a,b show the process of strain concentration for the sand specimen. In both figures, in the early stages of the test, the strain distribution is uniform along the whole surface. As it approaches the red line (OSL), the strains start to concentrate in one deformation plane. The third heat-map image shows the propagation of the deformation plane with the increase in strain after the OSL has been reached. Additionally, the heat map shows the created bifurcation angle as the result of the strain localization. The bifurcation angle of the sand with 0.5% XG was calculated as 65°, while the bifurcation angle of the sand with 1% XG was 75°. It can be concluded that the increase in biopolymer concentration can increase the bifurcation angle of sand. A similar trend was previously reported for the biopolymer-treated silty sand where the bifurcation angle was found through the analytical-numerical algorithm.

Observing Figure 11, a combination of the experimental response, numerical response and image-processing results for silty sand can be seen. Figure 11a represents the plain silty sand, Figure 11b represents the silty sand treated with 0.5% XG, and Figure 11c shows the silty sand with 1% GG. Even though the peak stress increased with the addition of biopolymers, the strain level for the OSL did not significantly change. This indicates that higher concentrations of XG and GG are required to postpone the OSL of the silty sand.

The heat maps in Figure 11a–c show the process of strain concentration for the biopolymer-treated silty sand. In all three figures, in the early stages of the test, the strain distribution is uniform along the specimens’ surfaces. As it approaches the OSL, the strains start to concentrate on one deformation plane. The second image shows the high strain concentration at the moment of the OSL, which was determined from the analytical-numerical algorithm. The third heat-map image shows the strain propagation that occurs after the OSL. Additionally, the heat maps show the created bifurcation angle due to the strain concentration. The bifurcation angle of the biopolymer-treated soil shows higher values for the biopolymer-treated silty sand. The plain silty sand (Figure 11a) had the bifurcation angle of 55°. The silty sand with 0.5% XG Figure 11b) had the bifurcation angle of 60°, while the concentration of 1% GG (Figure 11c) resulted in the bifurcation angle of 70°. As reported previously in this study, the bifurcation angle of the silty sand increased with the addition of biopolymers. The difference between the second heat-map image (at the OSL), and the third image (after the OSL) was more prominent in the sand than in the silty sand. The reason for this is the difference in the nature of the base material. The treated sand had a more brittle response than the silty sand. Furthermore, the break of the treated sand was cleaner, whereas the failure of the silty sand resulted in the partial crumbling of the material. Therefore, the nature of the treated sand was more suitable to observe using the image-processing software.

Observing the unconfined compression test using image-processing software showed high strain concentrations at the moment of the OSL. However, there are some drawbacks that should be considered. The image acquisition with a single camera and subsequent processing gave a 2D output only on one side of the specimen. However, during the actual 3D test, it was unknown on which specimen side the deformation band would occur. Therefore, the digitally obtained deformation planes for the tested specimens might not be the most critical ones. To solve these issues, multiple cameras facing each specimen side should be used.

## 5. Conclusions

The main objective was to perform the computational and experimental diagnostic strain-localization analyses in biopolymer-treated soil. Two types of soil (silty and clean sand) were investigated with the three types of biopolymers (xanthan gum, guar gum, and beta-glucan). A numerical-analytical algorithm was implemented to capture the stress-strain response and the inception of the strain localization. The experimental data were collected for the plain soil, and soil with 1, 2, and 4% biopolymer concentrations. Three strength tests were used for calibration. The experimental data of the silty sand with 2% biopolymers were used for validation purposes. 

The stress-strain response of the specimens subjected to uniaxial compression and triaxial stress states were simulated. In addition, the inception of the strain localization was calculated for each specimen and each stress state. It was found that the OSL was postponed during unconfined compression for most biopolymer concentrations, while it was advanced for the triaxial stress state. The peak stress in each test increased with the increase in biopolymer concentration, and the OSL always occurred close to the level of the peak stress. The bifurcation angle of silty sand increased with the increase in GG and BG concentration, but it started to decrease after adding more than 1% XG.

Several cube specimens of sand were tested for the unconfined compression and observed using image-processing software. The strain development was compared with the calculated OSL. The OSL appeared close to the peak stress, at which moment the image-processing software reported the initiation of the high strain concentrations. To capture more precise strain distributions and bifurcation angles with the image-processing software, a 3D image acquisition and analysis should be performed. That approach requires more equipment and will be utilized in future research. 

In conclusion, the diagnostic strain analysis shows that the presence of biopolymers in the soil can influence the onset of strain localization. Depending on the stress state, soil type, biopolymer type, and concentration, the strain localization can be postponed or advanced. Analyzing the complex mechanics of biopolymer-treated soil helps to understand their strength limitations. Understanding how biopolymers affect the strain localization and failure of soil materials can lead to their utilization in engineering practices.

## Figures and Tables

**Table 1:** Selected properties of the biopolymer-treated soil for the proposed model.

Material	E (MPa)	*β* (°)	ψ (°)	v
Plain soil	88	34	1	0.3
GG (1%)	234	63	1	0.3
GG (2%)	217	64	1	0.3
GG (4%)	184	65	1	0.3
BG (1%)	114	42	1	0.3
BG (2%)	130	46	1	0.3
BG (4%)	162	55	1	0.3
XG (1%)	249	54	1	0.3
XG (2%)	243	51	1	0.3
XG (4%)	233	46	1	0.3

## Data Availability

All data from this study can be provided by the corresponding author upon request.
